# Discrete but variable structure of animal societies leads to the false perception of a social continuum

**DOI:** 10.1098/rsos.160147

**Published:** 2016-05-11

**Authors:** Dustin R. Rubenstein, Carlos A. Botero, Eileen A. Lacey

**Affiliations:** 1 Department of Ecology, Evolution and Environmental Biology, Columbia University, New York, NY 10027, USA; 2 Center for Integrative Animal Behavior, Columbia University, New York, NY 10027, USA; 3 Department of Biology, Washington University in St Louis, St Louis, MO 63130, USA; 4 Museum of Vertebrate Zoology and Department of Integrative Biology, University of California, Berkeley, Berkeley, CA 94720, USA

**Keywords:** eusociality, cooperative breeding, sociality, social structure

## Abstract

Animal societies are typically divided into those in which reproduction within a group is monopolized by a single female versus those in which it is shared among multiple females. It remains controversial, however, whether these two forms of social structure represent distinct evolutionary outcomes or endpoints along a continuum of reproductive options. To address this issue and to determine whether vertebrates and insects exhibit the same patterns of variation in social structure, we examined the demographic and reproductive structures of 293 species of wasps, ants, birds and mammals. Using phylogenetically informed comparative analyses, we found strong evidence indicating that not all reproductive arrangements within social groups are viable in nature and that in societies with multiple reproductives, selection favours instead taxon-specific patterns of decrease in the proportion of breeders as a function of group size. These outcomes suggest that the selective routes to sociality differ depending upon whether monopolization of reproduction by one individual is possible and that variation within and among taxonomic groups may lead to the false perception of a continuum of social structures. Thus, the occurrence of very large societies may require either complete reproductive monopolization (monogyny/singular breeding) or the maintenance of a taxon-specific range of values for the proportional decrease in the number of breeders within a group (polygyny/plural breeding), both of which may reduce reproductive conflict among females.

## 1. Introduction

Reproductive altruism—most clearly manifests as the sacrifice of personal reproduction by some members of animal societies—represents a fundamental and enduring puzzle in biology. Complex altruistic societies occur in birds [1], mammals [2] and insects [3], as well as in a variety of other vertebrates [4] and invertebrates [5]. The term eusociality—meaning ‘true sociality’—was coined by Batra [6] and later expanded by Michener [7] and Wilson [3] to describe insect societies that share three characteristics: (i) overlapping generations, (ii) cooperative care of young, and (iii) reproductive division of labour (i.e. many individuals in a group are temporarily or permanently sterile). Although the same characteristics occur in avian and mammalian societies [8,9], studies of cooperative breeding in vertebrates and eusociality in insects have proceeded largely independently. A potential empirical bridge between vertebrate and insect sociality emerged with the report of eusociality in a diploid mammal, the naked mole-rat [10]. However, efforts to unify all social taxa under a single theoretical and terminological umbrella [11–13] have met with resistance, owing largely to differences in the biology of social vertebrates and insects and, in particular, the apparently exclusive occurrence of irreversibly sterile workers in eusocial insects ([14,15], but see [10]).

Kin selection is typically invoked to explain the evolution of altruistic societies [16]. Genetic relatedness is central to kin selection theory and high coefficients of relatedness among group members are thought to be essential to the occurrence of such societies [17]. One factor that contributes to the degree of kinship within social groups is the number of males with which a female mates. Specifically, theoretical analyses indicate that lifetime monogamy by females favours altruistic behaviour by increasing kinship among offspring [15,17,18]. This hypothesis is supported by comparative studies demonstrating that lifetime monogamy is ancestral in eusocial lineages of insects [19], and that the prevalence of multiple mating by individual females is reduced in cooperatively breeding birds [20] and mammals [21] relative to non-cooperative species.

Although the fundamental link between kin selection and altruistic behaviour is well established [22], the importance of lifetime monogamy is less universally accepted [23]. Other factors may also influence kinship and relatedness among offspring, in particular the number of breeding females per group [24,25]. Indeed, social species are typically divided into those characterized by a single breeding female per group (i.e. monogyny in insects, singular breeding in vertebrates) versus those with multiple breeding females per group (i.e. polygyny in insects, plural breeding in vertebrates) [1,2,26–28]. Although societies with one versus multiple breeding females have been described as distinct evolutionary outcomes that are shaped by different selective pressures [29], it has also been suggested that the structure of animal societies represents a continuum that ranges from completely egalitarian to completely despotic reproduction [11]. Distinguishing between these competing perspectives—discrete outcomes versus continuous variation in social structure—is fundamental to understanding the evolution of reproductive altruism in animals. Specifically, the existence of continuous variation would suggest that any ratio of breeders to non-breeders within a social group is possible and evolutionarily stable, while discrete outcomes would indicate that only a subset of values for the proportion of breeders within a group is possible and that selection favours social groups characterized by those particular values.

Here, we explore whether differences in demographic and reproductive structure (hereafter social structure) within and among disparate vertebrate and invertebrate lineages reflect continuous versus discrete variation in social behaviour. We collected data from the primary literature regarding the mean numbers of breeding (i.e. producing diploid offspring) and non-breeding females per group for 293 species of social wasps, ants, birds and mammals (electronic supplementary material, table S2). To align our analyses with standard terminology, we differentiated between species with only a single breeding female per group (i.e. monogyny or singular breeding) and those with more than one breeding female per group (i.e. polygyny or plural breeding). To evaluate the degree of continuity among these categories, we quantified how the proportion of breeding females to total females within a group (hereafter proportion of breeders) varies with the total number of females per group for societies with more than one reproductive female. Under a scenario of qualitatively distinct evolutionary outcomes that are shaped by different selective pressures, we would predict a significant negative relationship between the proportion of breeders and the total number of females in a group (indicating that a predictable ratio of breeders to non-breeders is favoured by selection [30]). By contrast, under a scenario of continuous variation in social structure, we would predict no relationship between the proportion of breeders and the total number of females in a group (i.e. species would occur anywhere in this parameter space). To control for shared evolutionary history, our analyses for each taxonomic group were phylogenetically informed using molecular trees compiled from the literature or assembled de novo for each lineage.

## 2. Material and methods

### 2.1. Trait data

A comprehensive search of the literature published through 2013 was conducted using Web of Knowledge (http://www.webofknowledge.com), Google Scholar (http://scholar.google.com) (search terms: group size, number of breeding females, number of breeding males, colony size, reproductive number, worker number, queen number, breeder number) and relevant edited volumes. Using the predominant pattern of reproductive behaviour observed in mature groups (*sensu* [31]), we identified each species as (i) monogynous or singular breeding if they were characterized by a mean of one breeding female per group, or (ii) polygynous or plural breeding if they were characterized by a mean of more than one breeding female per group. Although the degree of kinship among breeding females and the mechanisms of recruiting multiple breeders (e.g. primary polygyny, in which females of the same generation breed together versus secondary polygyny, in which female offspring remain to breed with their mother [31,32]) vary across species, the distinction between these two social structures represents a major division in how both social vertebrates [1,2] and eusocial insects [29,33] are categorized in the literature. Data for most species were derived from a single population; in the few cases, in which data from multiple populations were available, species averages were used for analysis. For each species, we sought to extract data on the numbers of breeding and non-breeding females per group from the same source. Since genetic data were unavailable for most species, breeding status was based upon social breeding roles. Because all of the polygynous ants in our dataset have castes but none of the polygynous wasps in our analyses display this attribute, we did not consider caste as a covariate in taxon-specific comparative analyses (see below). In addition, our analyses included only species for which both complete demographic information and data regarding phylogenetic relationships with other members of the same taxonomic group were available. Overall, our searches yielded data on the mean numbers of breeding and non-breeding females per group for 88 species of wasps, 140 species of ants, 30 species of birds and 35 species of mammals (electronic supplementary material, table S2).

Within-species variation in the number of breeding females per group occurs in both vertebrate and insect species. For example, temporal variation in queen number may arise as groups form and then mature (e.g. groups may be founded by multiple females, after which the number of breeders decreases to one as dominance hierarchies are established [32]). In addition, intraspecific variation in the number of breeding females per group—whether evolved or plastic in nature—may result from ecological factors [27,31], as observed by the transition between monogynous and polygynous ant colonies along ecological [34] and altitudinal gradients [35]. Despite the occurrence of such intraspecific variation in some species, however, our review of the literature suggests that group living taxa can be reliably assigned to one social structure or the other, at least within a given population [1,2,29,33].

We estimated the degree of monopolization of breeding positions as (1 – (no. breeding females/total females))/(1 – (1/total females)). This approach is qualitatively similar to the procedure employed by Duffy & MacDonald [36] to modify the eusociality index of Keller & Perrin [37] for use when only demographic data are available. Our index of monopolization of breeding positions ranged from 0 to 1: a value of 0 indicates that breeding is shared equitably among all females in the group, whereas a value of 1 indicates that breeding is limited to a single individual. The denominator in this formula accounts for the fact that the range of possible values changes as the number of individuals in the group changes. For all taxonomic groups, we included non-breeding females in our measures of total females because, despite evidence that genetic factors may influence caste determination in some species of eusocial insects [38], reproductive status in most species is based largely upon environmental factors. Thus, at a fundamental level, we acknowledge that all females are potential breeders at the time of birth or hatching. As a result, our metric provides a relative measure of the degree to which a given species achieves the potential for complete monopolization of breeding positions by a single female.

### 2.2. Estimation and use of phylogenies

Our phylogenetic hypothesis for mammals was derived from Bininda-Emonds *et al.* [39], for birds from Jetz *et al.* [40] and for ants from Moreau *et al.* [41] (see the electronic supplementary material). Because the ant phylogeny was resolved primarily to the level of genera, we added polytomies to the tips representing genera for which we had more than one species in our dataset (electronic supplementary material, figure S1*a*). Branch lengths for those polytomies were set to the average congeneric distance in Moreau *et al.* [41]; repeating these analyses with polytomy branch lengths set to both the maximum and minimum values of congeneric branch lengths in Moreau *et al.* [41] generated results that were qualitatively similar to those reported here. For wasps, our phylogenetic hypothesis (electronic supplementary material, figure S1*b*) was generated from nuclear and mitochondrial gene sequences downloaded from GenBank (http://www.ncbi.nlm.nih.gov/Genbank; accession numbers in the electronic supplementary material, table S1). These sequences were aligned using MUSCLE [42] and concatenated using Phyutility v. 2.2 [43]. A maximum-likelihood phylogeny for wasps was then generated with RAxML GUI v. 1.3.1 [44] using a GTR (General Time Reversible) model of nucleotide substitution, with the *Γ* model of rate heterogeneity and a set of multi-furcating constraints extracted from previously published phylogenies [45–48]. The resulting tree (electronic supplementary material, figure S1*b*) was ultrametrisized using Sanderson's non-parametric rate smoothing algorithm [49]. We added basal polytomies for genera for which genetic data for some species in our dataset were lacking using the same methods described for ants.

### 2.3. Comparative analyses

For polygynous and plural breeding species (i.e. those with more than one breeding female per group), we explored the relationship between the number of breeding females per group divided by the total number of females per group (i.e. proportion of breeders) versus the total number of females per group (see the electronic supplementary material). Importantly, a separate analysis was conducted for each of the four taxonomic groups included in our dataset using phylogenetically informed Bayesian regression models with uninformative priors and uniformly low levels of belief. Model chains were run via *MCMCglmm* in R [50] for 500 000 iterations with a burn-in of 200 000 iterations and thinning intervals of 100 iterations. To evaluate convergence, we assessed the mixing of Markov chain Monte Carlo (MCMC) chains visually [50] and computed formal diagnostics from Geweke [51] and Heidelberg & Welch [52] using the R-package ‘coda’ [53]. For each parameter in our models, we report the mean of the posterior distribution, the 95% credible interval (CI) and the MCMC *p*-value [50]. Comparable analyses were not conducted for monogynous and singular breeding species (i.e. societies with one breeding female) because, by definition, there is no variation in the number of reproductive females for these taxa.

## 3. Results

**Figure 1.**
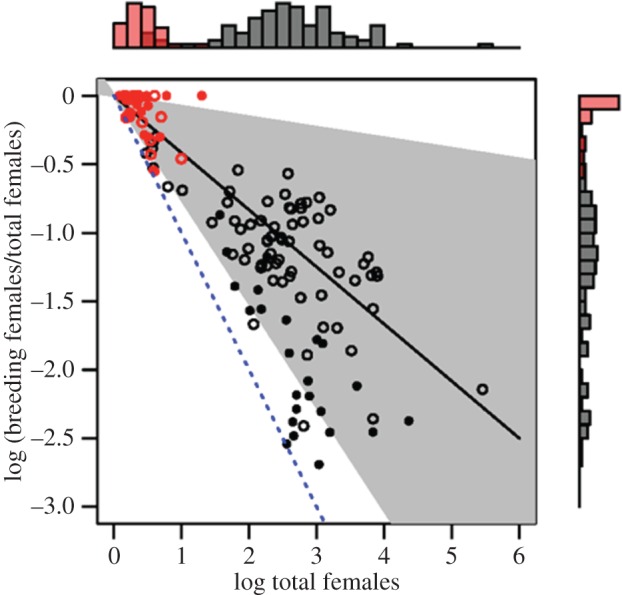
The relationship between the proportion of breeding to total females in a group versus the total number of females in a group for species with multiple breeding females per group in all taxonomic groups (wasps, black open circles; ants, black filled circles; birds, red open circles; mammals, red filled circles). Points are raw values; the line and shaded region represent respectively the regression line and associated 95% CIs for a phylogenetic general least-squares regression model. The dashed blue line indicates where monogynous/singular breeding species with one breeding female would fall. Histograms illustrate relative sampling densities for vertebrates (red bars) and insects (black bars). Sample sizes are provided in table 1.

Across all taxa considered, the proportion of breeders in societies with more than one reproductive female appeared to be dependent upon the total number of females in the group; when societies with multiple breeding females from all taxa (ants, wasps, mammals, birds) were pooled, we found that the proportion of breeders decreased significantly with the total number of females in the group (MCMCglmm with taxonomic class as a random variable: *β* (log total females) = −0.36, 95% CI = −0.42 to −0.28, *p* MCMC < 0.001; figure 1). This relationship, however, was only evident among groups containing more than approximately 10 females. Accordingly: (i) most vertebrates do not achieve the large group sizes in which this relationship becomes apparent, and (ii) the subset of insects that occur only in small groups exhibit the same pattern of variation in social structure observed in social vertebrates.

**Table 1.** Results of phylogenetically informed analyses examining the relationships between the proportion of breeding to total females in a group versus total females in a group for societies with multiple breeding females in wasp, ant, mammal and bird species.

	posterior mean	lower 95% CI	upper 95% CI	*p* MCMC
wasps (*n* = 71)	−0.378	−0.468	−0.290	<0.001
ants (*n* = 25)	−0.672	−0.774	−0.541	<0.001
birds (*n* = 14)^a^	−0.404	−0.622	−0.192	0.001
mammals (*n* = 20)	−0.206	−0.410	0.017	0.0733

a Average parameter estimates for 100 models with randomly selected trees from Jetz *et al.* [40].

**Figure 2.**
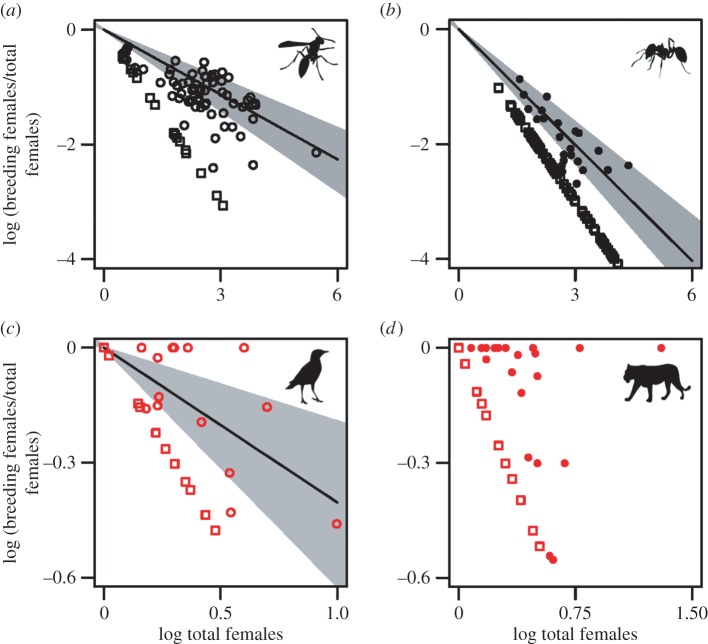
The relationship between the proportion of breeding to total females in a group versus the total number females in a group for (*a*) wasp, (*b*) ant, (*c*) bird and (*d*) mammal species with multiple breeding females per group (i.e. polygyny in insects and plural breeding in vertebrates; depicted as circles) or with one breeding female per group (i.e. monogyny in insects, singular breeding in vertebrates; depicted as squares). Points are raw values; lines and shaded regions (only plotted when significant associations were detected) represent respectively the regression lines and associated 95% CIs for phylogenetic general least-squares regression models. No CIs are presented for societies with one breeding female. Sample sizes are provided in table 1.

Separate phylogenetically informed analyses for each major taxonomic unit in our dataset (table 1) also revealed that the proportions of breeders decreased with the total number of females in the group in both insects and vertebrates (figure 2). The slopes of these relationships, however, differed among taxonomic units (table 1), with the proportion of breeders decreasing significantly as the total number of females in the group increased in wasps, ants and birds, and decreasing with marginal significance in mammals (figure 2). The pattern is least pronounced in mammals, presumably because: (i) mammalian group sizes are small relative to those in insects, and (ii) many of the mammalian species in our dataset live in societies in which all females reproduce (i.e. communal breeding). Importantly, this outcome suggests that in all taxa considered, societies characterized by multiple breeding females appear to be able to increase group sizes primarily through the maintenance of a predictable, relatively limited range of values for the proportion of breeding females within a group. In other words, there appears to be selection for taxon-specific relationships for how the proportion of breeders within a group declines with the total number of females per group. The distinction between societies with one and more than one breeding female—particularly at larger group sizes—suggests that the combination of evolutionary factors favouring reproductive sharing within groups are probably distinct from those that favour complete reproductive monopolization. More broadly, this result implies that rather than a continuum, there may be two distinct social trajectories among both eusocial insects and cooperatively breeding vertebrates: monogyny/singular breeding or polygyny/plural breeding, with the latter characterized by a limited, taxon-specific range of values for the proportion of breeders within a group. Thus, we suggest that the perception of a social continuum is probably a reflection of the observed variation within taxonomic groups (i.e. statistical noise), exacerbated by the lack of quantitative synthesis across widely disparate social taxa.

**Figure 3.**
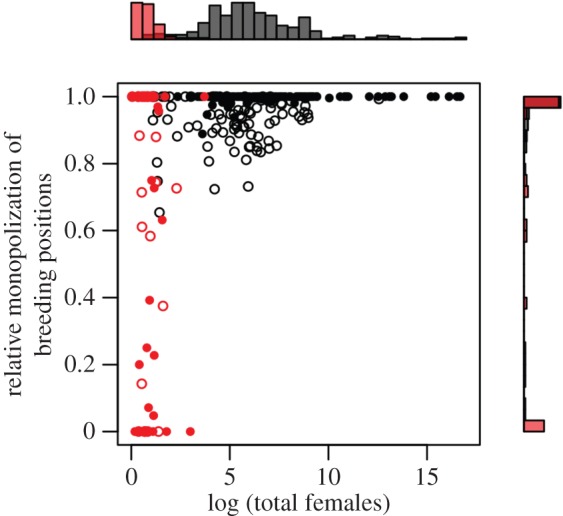
Relative monopolization of breeding positions as a function of group size. Vertebrates are depicted in red (open circles, birds (*n *= 30); filled circles, mammals (*n* = 35)) and insects in black (open circles, wasps (*n* = 88); filled circles, ants (*n* = 140)). Histograms depict standardized sampling densities for vertebrates (red bars) and insects (black bars). The relative monopolization of breeding positions ranges continuously from 0 to 1 in vertebrates but not in insects. In insects, the relative monopolization of breeding positions increases with the total number of females in a group.

The observed linear relationships between the proportion of breeding females and the total number of females per group suggest selection in both insects and vertebrates for a predictable and limited range of values for the proportion of breeders within a group. Based upon earlier findings [30], we predict that the quantitative value of this relationship (i.e. the slope of the associated regression) will depend upon the interplay between individual-level cooperation and conflict over reproductive opportunities. Ideally, this could be investigated by exploring taxonomic differences in patterns of reproductive skew (i.e. the variance in reproductive output among group members [54]). However, most indices of reproductive skew require information about not only the number of group members that breed, but also how reproduction is distributed among group mates [55]. Since the latter information is unavailable for most species in our dataset, we instead used a modified version of the eusociality index [37] to quantify the degree to which some members of a society are specialized for reproduction while others are specialized for non-breeding activities such as working or helping [36] (see Material and methods). The resulting index of reproductive monopolization varies from 0 (all individuals can potentially breed) to 1 (reproduction is monopolized by a single breeder). To avoid inflating differences among highly skewed breeding patterns in groups of different sizes, the index was standardized by dividing by the total number of individuals per group, meaning that groups with a single breeder were characterized by a value of one regardless of their overall size. Use of this index revealed that although vertebrates in our dataset exhibited the full range of possible values (from 0 to 1), all values for insects were above 0.5 (minimum values = 0.65 and 0.89 in wasps and ants, respectively; figure 3).

## 4. Discussion

Our analyses of eusocial insects and cooperatively breeding vertebrates indicate that in societies with more than one breeding female: (i) there is selection for a taxon-specific range of values for the proportion of breeders within a group, and (ii) these relationships are distinct from those for societies with only a single breeding female per group. Collectively, these findings suggest that the path to sociality differs when complete monopolization by one individual is possible versus when it is not. We propose that the appearance of a social continuum described in earlier studies (e.g. [11]) was driven by a focus on social species that do not achieve large group sizes (in which differences between social strategies are harder to detect) and by the variation within and among taxa in the relationship between the proportion of breeders and group size. This conclusion highlights: (i) the dichotomous nature of social structure within a diverse array of animal species, and (ii) the important role that group size plays in influencing social structure. Although group size has been proposed to be a key driver of social complexity in insects [16,56,57], its role in promoting differences in social structure among major taxonomic lineages has received little attention. Our analyses provide, to our knowledge, the first quantitative demonstration that group size (here, the total number of females in the group) is an important correlate of social structure both across disparate animal lineages and within socially diverse taxonomic groups.

Critically, our results demonstrate that not all possible social structures (i.e. proportions of breeding females per group) occur in nature. As the number of females in a group increases in polygynous and plural breeding societies, the percentage increase in the proportion of breeding females in each taxon changes in a predictable manner. This finding suggests that the evolution of larger groups may be fundamentally different when it is achieved by increasing numbers of both breeders and non-breeders (i.e. polygyny/plural breeding) versus increasing numbers of non-breeders only (i.e. monogyny/singular breeding). This hypothesis is consistent with data from bees indicating that polygynous and monogynous societies occur in different lineages as evolutionarily stable alternatives [29]. It is also consistent with studies of social organization and genetic architecture in ants, in which polygynous and monogynous colonies of the same species have distinct haplotypes whose loci occur together on a ‘social’ chromosome, a non-combining region of the genome [58,59].

One of the primary determinants of animal social structure is the interaction between ecology and the costs and benefits of group living [60,61]. In cooperatively breeding birds, variable climatic conditions may influence the formation of singular versus plural breeding groups [62]. In eusocial insects, ecology is thought to impact the adaptive consequences of having multiple breeding females per group [63]. For example, since insect societies with only one reproductive female are more susceptible to complete colony failure than those with multiple breeding females, the presence of multiple breeding females may be favoured in harsh or more unpredictable environments, where the higher likelihood of queen mortality puts entire colonies at risk [35,64]. However, because colonies with both types of social structures often co-occur in the same localities [34,35], it seems likely that ecological conditions interact with other factors to shape the social structure of a given population or species.

Another factor that may contribute to the distinction between monogynous/singular breeding and polygynous/plural breeding societies is variation in the ability of individuals to suppress reproductive competition from group mates [65,66]. Although reproductive conflict is expected to increase with increasing group size in species where non-breeders are capable of reproducing [25], allowing some group mates to share in reproduction may increase the ability of dominant animals to suppress subordinates in situations in which one individual cannot fully monopolize reproduction [30]. This hypothesis predicts: (i) selection for a limited subset of values for the proportion of breeders in societies with more than one reproductive female [30], and (ii) variation in these relationships among taxa that differ in their ability to suppress reproduction in conspecifics. Both of these predictions are supported by our analyses. Potential mechanisms of reproductive suppression that may contribute to maintaining a predictable proportion of breeders include physical aggression and threat displays by dominants [67,68] as well as pheromonal cues of dominance, health and reproductive capacity [69–71].

The causal bases for the taxonomic differences in relationships among group size, reproductive monopolization and social structure reported here remain unknown. To understand this variation, we suggest that at least two lines of inquiry deserve further attention. First, fundamental life-history differences may differentially influence patterns of sociality in vertebrates and insects. For example, because body sizes for vertebrates are larger than those for insects, it is possible that group size in the former may be more strongly limited by the number of individuals that can occupy a territory or nest site. At the same time, female vertebrates tend to be more constrained in the number of young that they can produce. Second, pronounced differences in ontogenetic patterns may limit the relative ability of vertebrates to monopolize reproduction. Specifically, vertebrates lack the free-living larval stage during which reproductive control occurs in most insects [16,72] and this ontogenetic difference may limit the ability of the former to monopolize reproduction, thereby altering the selective pressures favouring the evolution of very large monogynous social groups. These potential differences among taxa occur within the larger context of similar ecological pressures acting on vertebrates and insects that may influence group size and thus contribute to variation in social structure, both within and among taxonomic units. Consistent with this hypothesis, limitation of suitable habitats or breeding sites can constrain dispersal and thus favour group formation in both vertebrates [60,73] and insects [63]. Similarly, temporally variable and climatically unpredictable environments may favour cooperative breeding in vertebrates [74–76] and polygyny in insects [35,64].

To conclude, we suggest that ongoing debate regarding the nature of variation among animal social structures—specifically, whether such variation reflects a continuum of reproductive options versus distinct alternative social strategies—may have been driven in part by a lack of taxonomic synthesis. By comparing quantitative data from ants, wasps, birds and mammals, we have shown that not all possible social structures—defined as the proportion of breeding females to the total number of females in a group—occur among natural groups of social animals. Although the same general relationships between the proportion of female breeders and the total females per group are observed in all animal lineages considered here, the slopes of these relationships and the range of group sizes differ among taxa, suggesting that each one of these clades is characterized by a different balance between numbers of breeders and non-breeders and may be subject to different constraints. Despite clear differences between societies with one versus more than one breeding female per group, the typical variability observed in measurements of social structures within and among taxonomic groups can easily lead to a false perception of a social continuum. Thus, our data suggest that the occurrence of very large societies may require either complete reproductive monopolization (i.e. monogyny/singular breeding) or a taxon-specific range of values for the proportional decrease in the number of breeders within a group (i.e. polygyny/plural breeding), both of which may reduce reproductive conflict among female group mates [30]. Collectively, our analyses reveal previously undetected patterns of social structure that yield important insights into potential links between group size, social structure and taxonomic diversity in social organization.

## Supplementary Material

Rubensteinetal_SuppMat

## Supplementary Material

Table S2

## Data accessibility

All phylogenetic hypotheses and raw data are provided in the electronic supplementary material.
