# Supplementary material for: Discrete but variable structure of animal societies leads to the false perception of a social continuum
Source: R Soc Open Sci. 2016 May 11;3(5):160147. doi: 10.1098/rsos.160147 (PMC4892458; doi:10.1098/rsos.160147)
Supplement: Rubensteinetal_SuppMat [file rsos160147supp1.doc]

**Phylogenies**

Molecular phylogenies of ants and wasps are provided in NEWICK format and as trees (Figure S1):

***Ants***

((((((((((Wasmannia_auropunctata:80.543597,(Myrmica_gallienii:32.200003,Myrmica_limanica:32.2,Myrmica_rubra:32.2,Myrmica_ruginodis:32.2,Myrmica_rugulosa:32.2,Myrmica_sabuleti:32.2,Myrmica_scabrinodis:32.2,Myrmica_schencki:32.2,Myrmica_sulcinodis:32.2):48.3436):14.914481,Megalomyrmex_mondabora:95.458079):3.039398,(((Tetramorium_caespitum:81.274833,Crematogaster_dohrni:81.274833):13.206433,(((Temnothorax_acervorum:32.2,Temnothorax_ambiguus:32.2,Temnothorax_curvispinosus:32.2,Temnothorax_longispinosus:32.2,Temnothorax_muscorum:32.2,Temnothorax_nylanderi:32.2,Temnothorax_unifasciatus:32.2):49.97609,Solenopsis_invicta:82.176092):6.70309,Monomorium_pharaonis:88.879182):5.602084):2.136727,(((Aphaenogaster_barbara:32.20001,Aphaenogaster_rudis:32.2,Aphaenogaster_treatae:32.2):46.77593,Messor_barbara:78.975934):15.094398,(Pogonomyrmex_subnitidus:32.200008,Pogonomyrmex_badius:32.2,Pogonomyrmex_californicus:32.2,Pogonomyrmex_montanus:32.2,Pogonomyrmex_occidentalis:32.2,Pogonomyrmex_rugosus:32.2,Pogonomyrmex_subnitidis:32.2):61.87033):2.54766):1.879484):1.330753,((Pyramica_emmae:92.102264,((Cyphomyrmex_cornutus:32.2,Cyphomyrmex_rimosus:32.2):29.02623,((Atta_cephalotes:32.2,Atta_sexdens:32.2,Atta_texana:32.2):12.79853,Trachymyrmex_urichi:44.998535):16.2277):30.876029):1.713836,(Pheidole_harrisonfordi:32.200006,Pheidole_dentata:32.2,Pheidole_embolopyx:32.2,Pheidole_multispina:32.2,Pheidole_nigricula:32.2,Pheidole_rudia:32.2,Pheidole_rugiceps:32.2,Pheidole_specularis:32.2):61.6161):6.012129):15.964119,(((((Cataglyphis_bicolor:32.2,Cataglyphis_cursor:32.2):24.88054,(Formica_pallidefulva:32.20001,Formica_foetens:32.20001,Formica_fusca:32.2,Formica_japonica:32.2,Formica_nitidiventris:32.2,Formica_pallidefulva_schaufussi:32.2,Formica_podzolica:32.2,Formica_pratensis:32.2,Formica_rufa:32.2,Formica_yessensis:32.2):24.88054):18.630451,Oecophylla_longinoda:75.710996):4.91132,(Polyrhachis_hodgsoni:44.1,(Camponotus_christi_ferrugineus:32.200005,Camponotus_chromaiodes:32.2,Camponotus_ferrugeneus:32.2,Camponotus_herculeanus:32.2,Camponotus_laevigatus:32.2,Camponotus_modoc:32.2,Camponotus_noveboracensis:32.2,Camponotus_pennsylvanicus:32.2,Camponotus_socius:32.2,Camponotus_vicinus:32.2):11.9):36.522316):5.605775,(Prenolepis_imparis:77.790399,(Lasius_carniolicus:32.2,Lasius_flavus:32.2,Lasius_neglectus:32.2,Lasius_niger:32.2):45.5904):8.437692):29.564258):1.963163,((((Rhytidoponera_chalybaea:32.2,Rhytidoponera_confusa:32.2,Rhytidoponera_purpurea:32.2):39.05885,Ectatomma_tuberculatum:71.258849):8.275893,(Gnamptogenys_bicolor:32.2,Gnamptogenys_hartmani:32.2,Gnamptogenys_moelleri:32.2,Gnamptogenys_striatula:32.2):47.33474):20.199944,Heteroponera_relicta:99.734686):18.020825):3.323845,(Iridomyrmex_purpureus:84.080404,Dolichoderus_cuspidatus:84.08041):36.998952):1.899633,(((Pseudomyrmex_termitarius:32.2,Pseudomyrmex_triplarinus:32.2):18.25178,Tetraponera_sp.:50.451783):57.839604,(Myrmecia_dispar:32.2,Myrmecia_froggatti:32.2,Myrmecia_mandibularis:32.2,Myrmecia_nigrocincta:32.2,Myrmecia_picta:32.2,Myrmecia_pilosula:32.2,Myrmecia_varians:32.2,Myrmecia_vindex:32.2):76.09139):14.687602):1.767526,((Dorylus_anomma:32.2,Dorylus_wilverthi:32.2):61.77191,((Eciton_burchelli:32.2,Eciton_hamatum:32.2,Eciton_rapax:32.2):11.98626,(Labidus_praedator:28.144305,Neivamyrmex_nigrescens:28.144305):16.041957):49.785648):30.774606):7.298617,((((Platythyrea_lamellosa:32.2,Platythyrea_schultzei:32.2):78.49864,(((((Cryptopone_sauteri:57.520963,(Pachycondyla_tarsata:32.200007,Pachycondyla_sublaevis:32.200007,Pachycondyla_sp.:32.200007,Pachycondyla_sennaarensis:32.200007,Pachycondyla_marginata:32.200007,Pachycondyla_havilandi:32.200007,Pachycondyla_excavata:32.200007,Pachycondyla_australis:32.200007,Pachycondyla_analis:32.2,Pachycondyla_apicalis:32.2,Pachycondyla_australis_:32.2,Pachycondyla_Bothroponera_sp.:32.2,Pachycondyla_Bothroponera_sublaevis:32.2,Pachycondyla_caffraria:32.2,Pachycondyla_foetens:32.2,Pachycondyla_Hagensia_havilandi:32.2,Pachycondyla_obscuricornis:32.2,Pachycondyla_senaarensis:32.2,Pachycondyla_villosa:32.2,Pachycondyla_wroughtoni:32.2):25.32096):17.763593,(Diacamma_ceylonense:32.2,Diacamma_cyaneiventre:32.2,Diacamma_rugosum:32.2,Diacamma_sp.:32.2):43.08456):5.449463,(Hypoponera_excavata:32.2,Hypoponera_gleadowi:32.2):48.53402):4.740883,(((Dinoponera_australis:32.2,Dinoponera_quadriceps:32.2):38.88267,(Leptogenys_ocellifera:32.200004,Leptogenys_attenuata:32.2,Leptogenys_castanea:32.2,Leptogenys_diminuta:32.2,Leptogenys_distinguenda:32.2,Leptogenys_kraepelini:32.2,Leptogenys_mjobergi:32.2,Leptogenys_nitida:32.2,Leptogenys_schwabi:32.2):38.88267):9.409988,((Anochetus_bequaerti:32.2,Anochetus_faurei:32.2,Anochetus_katonae:32.2):18.24041,(Odontomachus_haematodus:32.200006,Odontomachus_bauri:32.2,Odontomachus_haematoda:32.2):18.24041):30.052246):4.982244):4.505402,Centromyrmex_bequaerti:89.980305):20.718339):12.332277,Paraponera_clavata:123.03092):5.142035,(((Prionopelta_amabilis:80.1498,Onychomyrmex_hedleyi:80.149799):40.221156,(Amblyopone_pluto:32.2,Amblyopone_reclinata:32.2,Amblyopone_sp.:32.2):88.17095):5.134408,(Proceratium_goliath:32.2,Proceratium_itoi:32.2):93.30536):2.667592):3.872179);

***Wasps***

(((Parischnogaster_striatula:0.3708280701,Parischnogaster_nigricans:0.3708280701,Parischnogaster_jacobsoni:0.3708280701,Parischnogaster_alternata:0.3708280701,Parischnogaster_mellyi:0.3708280701):0.2490833559,(Eustenogaster_sp._1:0.3542709656,(Liostenogaster_sp.:0.2305510804,Liostenogaster_flavolineata:0.2305510804,Liostenogaster_vechti:0.2305510804):0.1237198851):0.2656404605):0.3800885739,((((((Polistes_annularis:0.1754707071,Polistes_exclamans:0.1754707071):0.1638889528,(Polistes_bellicosus:0.1722715479,Polistes_dorsalis:0.1722715479):0.167088112):0.09517166354,Polistes_olivaceus:0.4345313235):0.160853657,((((((Chartergus_chartarius:0.1205985546,((Synoeca_septentrionalis:0.01716507167,Synoeca_surinama:0.01716507167):0.03902819996,((Polybia_velutina:0.05116050538,Polybia_singularis:0.05116050538,Polybia_simillima:0.05116050538,Polybia_scutellaris:0.05116050538,Polybia_quadricincta:0.05116050538,Polybia_platycephala_sylvestris:0.05116050538,Polybia_platycephala:0.05116050538,Polybia_paulista:0.05116050538,Polybia_parvulina:0.05116050538,Polybia_micans:0.05116050538,Polybia_gorytoides:0.05116050538,Polybia_erythrothorax:0.05116050538,Polybia_dimidiata:0.05116050538,Polybia_chrysothorax:0.05116050538,Polybia_catillifex:0.05116050538,Polybia_bistriata:0.05116050538,Polybia_bicyttarella:0.05116050538,(Polybia_velutina:0.01716658817,(Polybia_sericea:0.01716507167,Polybia_emaciata:0.01716507167):1.51649594e-06):0.03399391721,((((Polybia_ignobilis:0.02480156091,(Polybia_scrobalis:0.02147438356,Polybia_ruficeps:0.02147438356):0.00332717735):0.001120174344,Polybia_occidentalis:0.02592173525):0.002192810358,(Polybia_striata:0.02514476075,Polybia_rejecta:0.02514476075):0.002969784854):0.005619482184,Polybia_jurinei:0.03373402779):0.01742647759):0.001549787773,(Metapolybia_cingulata:0.01716507167,Metapolybia_azteca:0.01716507167):0.03554522148):0.003482978483):0.06440528298):0.104419084,((Brachygastra_scutellaris:0.0815666955,Brachygastra_bilineolata:0.0815666955,Brachygastra_augusti:0.0815666955,Brachygastra_mellifica:0.0815666955):0.08057511242,(Protopolybia_surinama:0.08150370023,Protopolybia_minutissima:0.08150370023,Protopolybia_acutiscutis:0.08150370023,Protopolybia_scutellaris:0.08150370023,(Protopolybia_sedula:0.06472579962,Protopolybia_exigua:0.06472579962):0.01677790061):0.0806381077):0.06287583073):0.1019208808,(((Pseudopolybia_compressa:0.01716507167,Pseudopolybia_difficilis:0.01716507167):0.09932304527,Pseudopolybia_vespiceps:0.1164881169):0.08781065653,(Chartergellus_communis:0.01716507167,Chartergellus_atectus:0.01716507167):0.1871337018):0.122639746):0.02753928137,(Parachartergus_colobopterus:0.171994074,Parachartergus_fraternus:0.171994074):0.1824837268):0.07405533244,(Angiopolybia_pallens:0.2779110823,((Agelaia_vicina:0.01716507167,Agelaia_lobipleura:0.01716507167,Agelaia_fulvofasciata:0.01716507167,Agelaia_areata:0.01716507167,Agelaia_cajennensis:0.01716507167):0.1323636278,(Apoica_gelida:0.06532868531,Apoica_pallens:0.06532868531):0.08420001421):0.1283823827):0.150622051):0.1015994307,(Ropalidia_trichopthalma:0.01716507167,Ropalidia_taiwana_koshuensis:0.01716507167,Ropalidia_romandi:0.01716507167,Ropalidia_montana:0.01716507167,Ropalidia_marginata:0.01716507167,Ropalidia_mackayensis:0.01716507167,Ropalidia_cristata:0.01716507167,Ropalidia_kurandae:0.01716507167):0.5129674923):0.06525241649):0.1150077464,Polybiodes_tabidus:0.7103927268):0.124156004,((Vespa_crabro:0.3667422117,(Vespa_affinis_indosinensis:0.1885332636,Vespa_analis:0.1885332636,(Vespa_simillima:0.1171440734,Vespa_mandarinia:0.1171440734):0.07138919022):0.1782089481):0.1953255431,(Provespa_anomala:0.3371720351,(Dolichovespula_maculata:0.1972585832,(Vespula_maculifrons:0.1378342312,Vespula_squamosa:0.1378342312):0.05942435199):0.1399134518):0.2248957197):0.2724809761):0.1654512691);

**Figure S1**. Molecular phylogenies of (A) ants and (B) wasps.

(A) Ants

(B) Wasps

**Table S1**. GenBank accession numbers for sequences used to build the wasp phylogeny.

| Latin Name | Gene | | | | | |
| --- | --- | --- | --- | --- | --- | --- |
|  | COI | 28S | 18S | 16S | RNA Polymerase II | 12S |
| *Agelaia^A^* | GU596948.1 | GU596702.1 |  | GU596686.1 |  | GU596571.1 |
| *Angiopolybia pallens* | AY382248.1 |  |  |  |  |  |
| *Apoica gelida* | AY918919.1 |  |  |  |  |  |
| *Apoica pallens* | AY918921.1 | GU596707.1 |  | GU596614.1 |  |  |
| *Brachygastra augusti* | AY382253.1 |  |  |  |  |  |
| *Brachygastra mellifica* | AY382254.1 |  |  |  |  |  |
| *Chartergellus communis* | GU596902.1 | GU596721.1 |  | GU596689.1 |  |  |
| *Chartergus^B^* | AY918911.1 |  |  |  |  |  |
| *Dolichovespula maculata* | GU596843.1 | EU367175.1 | EF190707.1 | GU596694.1 | EF190793.1 |  |
| *Eustenogaster^C^* |  | EF190755.1 | EF190725.1 | AF066918.1 | EF190811.1 |  |
| *Liostenogaster flavolineata* | GU596839.1 | GU596730.1 | EF190726.1 | AF066939.1 | EF190812.1 | GU596568.1 |
| *Liostenogaster vechti* | GU596840.1 | GU596731.1 |  | AF066917.1 |  |  |
| *Metapolybia cingulata* | GU596904.1 | GQ374720.1 | GQ410613.1 | GU596623.1 | EF190817.1 | GU596575.1 |
| *Parachartergus colobopterus* | AY382249.1 |  |  |  |  |  |
| *Parachartergus fraternus* | AY382250.1 | EF190762.1 | EF190732.1 |  | EF190818.1 |  |
| *Parischnogaster alternata* |  | AF066928.1 |  | AF066938.1 |  |  |
| *Parischnogaster mellyi* | GU596841.1 | GU596752.1 | EF190727.1 | GU596697.1 | EF190813.1 | GU596523.1 |
| *Polistes annularis* | EF136417.1 | GU596754.1 | X74762.1 | GU596639.1 |  | GU596524.1 |
| *Polistes bellicosus* | EF136421.1 | GU596758.1 |  | GU596642.1 |  | GU596569.1 |
| *Polistes dorsalis* | EF136435.1 | GU596770.1 |  | GU596652.1 |  | GU596532.1 |
| *Polistes exclamans* | EF136437.1 | GU596771.1 |  | GU596693.1 |  | GU596533.1 |
| *Polistes olivaceus* | EF136450.1 |  |  |  |  |  |
| *Polybia emaciata* | AY382257.1 | GU596795.1 |  | GU596674.1 |  | GU596582.1 |
| *Polybia ignobilis* | AY382256.1 |  |  |  |  |  |
| *Polybia jurinei* | GU596928.1 | GU596797.1 |  |  |  |  |
| *Polybia occidentalis* | GU596930.1 | GU596799.1 |  | GU596678.1 |  | GU596587.1 |
| *Polybia rejecta* | GU596933.1 |  |  |  |  |  |
| *Polybia ruficeps* | GU596934.1 |  |  |  |  |  |
| *Polybia scrobalis* | AY382261.1 |  |  |  |  |  |
| *Polybia sericea* | AY918913.1 | GU596801.1 |  | GU596679.1 |  |  |
| *Polybia striata* | GU596936.1 |  |  | GU596680.1 |  | GU596594.1 |
| *Polybia velutina* |  | GU596802.1 |  |  |  |  |
| *Polybioides* | GU596937.1 |  |  | GU596681.1 |  | GU596583.1 |
| *Protopolybia exigua* | GU596940.1 |  |  | GU596684.1 |  | GU596565.1 |
| *Protopolybia scutellaris* | GU596941.1 | GU596805.1 |  | GU596685.1 |  | GU596593.1 |
| *Protopolybia sedula* | GU596942.1 |  |  |  |  |  |
| *Provespa anomala* | AB585952.1 | GU596806.1 |  | GU596609.1 |  | GU596550.1 |
| *Pseudopolybia compressa* | GU596945.1 | GU596808.1 |  | GU596699.1 |  | GU596553.1 |
| *Pseudopolybia vespiceps* | GU596944.1 | GU596809.1 |  |  |  | GU596554.1 |
| *Ropalidia^D^* | AY918909.1 | EF190765.1 | EF190735.1 |  | EF190821.1 |  |
| *Synoeca septentrionalis* | AY382263.1 |  |  |  |  |  |
| *Vespa analis* | AB585948.1 |  |  |  |  |  |
| *Vespa crabro* | KC136020.1 | AF067145.1 |  |  |  |  |
| *Vespa mandarinia* | GU596946.1 | GU596813.1 | AB126809.1 | GU596700.1 |  | GU596570.1 |
| *Vespa simillima* | HM180937.1 |  |  |  |  |  |
| *Vespula maculifrons* | AF142537.1 | GU596816.1 | EF190708.1 |  | EF190794.1 | GU596559.1 |
| *Vespula squamosa* | EF136416.1 | GU596817.1 | EF190730.1 | GU596701.1 | EF190816.1 | GU596560.1 |
| *Myrmica incompleta* (ant outgroup) | FJ824434.1 | DQ353629.1 | DQ353432.1 |  |  | EF518764.1 |

^A^ sequences from *Agelaia pallipes*

^B^ sequences from *Chartergus metanotalis*

^C^ 28S, 18S and RNA Polymerase II sequences from *Eustenogaster* *calyptodoma*; 16S sequence from *Eustenogaster* *fraterna*

^D^ 28S, 18S and RNA Polymerase II sequences from *Ropalidia opifex*; COI sequence from *Ropalidia KMP-2004*

**Table S2**. Raw trait data.

See Excel file
